# Redesigning Enzymes for Biocatalysis: Exploiting Structural Understanding for Improved Selectivity

**DOI:** 10.3389/fmolb.2022.908285

**Published:** 2022-07-22

**Authors:** Yaoyu Ding, Gustavo Perez-Ortiz, Jessica Peate, Sarah M. Barry

**Affiliations:** Department of Chemistry, Faculty of Natural, Mathematical and Engineering Sciences, King’s College London, London, United Kingdom

**Keywords:** biocatalysis, enzymes, protein engineering, structural biology, regioselectivity, stereoselectivity, rational design

## Abstract

The discovery of new enzymes, alongside the push to make chemical processes more sustainable, has resulted in increased industrial interest in the use of biocatalytic processes to produce high-value and chiral precursor chemicals. Huge strides in protein engineering methodology and *in silico* tools have facilitated significant progress in the discovery and production of enzymes for biocatalytic processes. However, there are significant gaps in our knowledge of the relationship between enzyme structure and function. This has demonstrated the need for improved computational methods to model mechanisms and understand structure dynamics. Here, we explore efforts to rationally modify enzymes toward changing aspects of their catalyzed chemistry. We highlight examples of enzymes where links between enzyme function and structure have been made, thus enabling rational changes to the enzyme structure to give predictable chemical outcomes. We look at future directions the field could take and the technologies that will enable it.

## 1 Introduction

Enzymes are of interest in chemical synthesis as they catalyze a wide array of chemical transformations (e.g., C–H bond activation, cyclization, and stereospecific reduction) which are extremely challenging, using even modern small-molecule catalysis. Furthermore, enzymes carry out these reactions often with unrivaled chemo-, regio-, and stereoselectivity and under mild conditions. The drive to make synthesis more sustainable has led to a rapid expansion in the use of enzymes in industry and is facilitated by improved availability of reliable off-the-shelf biocatalysts and screening methodologies (Hughes and Lewis, 2018). Several factors have influenced the availability of biocatalysts. The vast increase in sequence data has allowed genome mining for enzymes with specific domains which give rise to known functionalities, e.g., alcohol dehydrogenases, cytochrome P450s, etc. Improvements in cloning technology and construct optimization, as well as an array of expression strains and solubility tags, have given protein scientists an impressive toolbox to optimize the production of previously incalcitrant proteins. Additionally, cofactor recycling systems have made biocatalytic reactions that were unviable due to expensive enzyme cofactors, e.g., S-adenosylmethionine and NADPH consumed in reactions, now more economical. However, going forward, to expand biocatalysis for sustainable chemical synthesis, methods and understanding are needed to harness enzymes with desired functionality to replace and complement existing synthetic strategies. This can be accomplished through a combination of enzyme discovery and protein engineering. In fact, future developments in protein engineering are likely to have the most profound impact on industrial biocatalysis (Bell et al., 2021).

Engineering enzymes can be carried out via rational design, e.g., targeting specific domains, conserved motifs, creating fusion proteins, or via non-rational methods such as directed evolution (Arnold, 2018) (Yang et al., 2019) (Ali et al., 2020) (Lovelock et al., 2022). Directed evolution strategies have already been successfully applied to the industrial production of high-value molecules, for example, the use of a transaminase to produce sitagliptin by Merck and Codexis (Savile et al., 2010). Transaminases are pyridoxyl phosphate-dependent enzymes that utilize an aldehyde or ketone, and an amine to catalyze the chemical equivalent of a stereospecific reductive amination. This is an increasingly important method to make chiral amines in industrial biocatalysis (Kelly et al., 2020). To introduce a key chiral amine in sitagliptin the transaminase was subjected to directed evolution. The resulting engineered enzyme contains five mutations around the active site to accommodate the large tri-fluorophenyl group on pro-sitagliptin (Savile et al., 2010).

However, directed evolution strategies, while extremely powerful, require high throughput methods to screen thousands of mutants and usually an operationally simple colorimetric assay, to select positive hits (Wang et al., 2021). These assays can be difficult to optimize and are usually designed to detect only the desired outcome. This could miss unpredicted, but useful changes in catalyzed chemistry (Hubert and Barry, 2016). Semi-rational design requires an enzyme structure or homology model, enabling the identification of key enzyme residues or motifs, and employs various strategies such as site saturation mutagenesis, Combinatorial Active-Site Saturation Test (CAST), and Iterative Saturation Mutagenesis (ISM) (Wang et al., 2017). These strategies can also generate hundreds of mutants and screening is a common bottleneck. Rational engineering generally involves targeting a limited number of residues in a binding pocket, consensus sequence, or motif. Thus, it produces fewer mutants that can be screened using low-medium throughput methods such as High performance liquid chromatography (HPLC). Chromatographic screening methods gather more information, such as unexpected products and the regio and stereoselectivity of the reaction, which is of vital importance in synthesis. But, while high throughput HPLC methodology has improved, it is comparatively expensive. However, rational mutation relies on significant knowledge of the structure and mechanism of the enzyme.

Here, we discuss rational or semi-rational protein engineering approaches to alter aspects of enzyme chemistry with a focus on improving stereo- and/or regioselectivity. We briefly discuss expanding substrate tolerance. These approaches help to provide insight into enzyme structure-activity relationships and highlight gaps in our understanding. The following is not intended to be an exhaustive review. We focus on approaches which are guided by, or can be explained through, structural and mechanistic understanding of the enzyme. We have also selected examples to illustrate each approach, focusing on enzymes already used in industrial contexts or transformations of particular interest in organic synthesis (Winkler et al., 2021) (Marshall et al., 2021).

## 2 Rational Mutagenesis

Stereoselective and regioselective reactions are vital for the successful chemical synthesis of complex molecules. However, many stereoselective reactions are catalyzed by heavy metal-containing small-molecule catalysts which can be toxic. The benefit of small-molecule catalysts is that they can be designed to produce one or the other enantiomer selectively.

Enzymes can carry out transformations under mild conditions avoiding the need for heavy metals and solvents, and thus offer a path to more sustainable chemistry. Most enzymes also afford a single stereo or regioisomer. Should this not be the desired isomer of a given chemical target, a complementary enzyme may need to be identified. In other cases, enzymes produce mixtures of products which then require time-consuming separation. Both problems could be solved by a bioinformatics approach, i.e., searching genetics databases for similar enzymes, followed by extensive production and screening of candidate enzymes. This is time-consuming and success is not guaranteed. For many enzyme families, predicting the substrate and chemistry based on sequence or homology model alone is still difficult. For this reason, many efforts have been made to develop selective biocatalysts via rational protein engineering.

Rational mutagenesis can involve strategies of differing complexity. Sequence alignment and sufficient knowledge of conserved motifs can often provide sufficient guidance. However, for enzymes that undergo substantial conformational change on substrate binding, crystal structure data, homology modelling, and substrate docking studies may be required to produce a more detailed picture. Detailed information about the enzyme mechanism could also be necessary. Here we discuss several rational engineering strategies to alter enzyme reaction outcomes. They include, mutating residues directly involved in substrate binding interactions to alter substrate orientation, change steric crowding in the active site to promote altered regio- and stereoselectivity and improved enzyme promiscuity, and also mutating residues directly involved in catalysis.

### 2.1 Analysis of Protein Sequence and Structure to Aid Protein Engineering

Structurally characterized enzymes, especially with substrate and/or cofactors bound, offer the best opportunities for rational design. However, identifying where mutations should be introduced, is enzyme or enzyme family dependent, as the structural drivers of stereo and regioselectivity will differ. Furthermore, many enzymes cannot be structurally characterized in the presence of substrate because the enzyme turns over the substrate and appropriate inert substrate analogs may not be available. Key information about substrate orientation, active site interactions, and conformational change resulting from ligand binding will thus be missing. Thus, computationally docking substrates into crystal structures or even homology models can be a powerful tool enabling rational protein engineering. For example, the plant cytochrome P450 (CYP72A63) oxidizes the terpene 11-oxo-β-amyrin producing a mixture of oxidized products. The reaction occurs at a single carbon and the enzyme sequentially oxidizes the carbon in a series of two-electron oxidations from C-H to alcohol, aldehyde, and finally a carboxylic acid (Sun et al., 2020). Cytochrome P450s are heme-dependent enzymes that utilize molecular oxygen to facilitate a wide variety of oxidative transformations on a vast array of substrates (Greule et al., 2018). Despite functional and primary sequence diversity, their tertiary structures are highly conserved and contain several key motifs which significantly influence catalysis (Poulos, 2014). Docking studies of CYP72A63 revealed just three residues responsible for chemo- and regio-selectivity (Sun et al., 2020). It appeared that the methyl group of T338, in a conserved motif located in the I-helix above the heme, reduced the enzyme’s affinity for the partially oxidized intermediates (alcohol and aldehyde). This resulted in intermediate release and prevented further oxidation to the acid. The T338S mutant decreased hydrophobicity in the active site resulting in retention of the intermediate and thus production of the carboxylic acid, glycyrrhetinic acid. Furthermore, a single mutation L398I rotated 11-oxo-β-amyrin and relocated C-29 close to the heme-Fe center (Sun et al., 2020). Cytochrome P450s are of growing interest as biocatalysts due to their potential as C-H bond activation catalysts as illustrated in the example above (Zhang et al., 2019). The ability to activate inert C-H bonds selectively, even on complex substrates, is considered a holy grail of synthetic chemistry and an important green chemistry target (Arndtsen et al., 1995) (Dalton et al., 2021). There are however still significant gaps in our understanding of the relationship between P450 structure and function.

Identifying conserved residues or motifs in an enzyme is also enabled by easy access to the large bank of sequence data and bioinformatic tools. This information is vital for “preengineering”. For example, in an imine reductase G-36 (IRED), N121 and S260 were identified from the docking model as targets to enable expansion of the binding cavity. Additional target residues were identified from a sequence alignment of 70 IREDs. Thus, just seven variants were screened and the catalytic efficiency was improved ten times with a near-perfect stereoselectivity (R, 99% enantiomeric excess (ee)) (Zhang et al., 2022). Equally, in the transaminase from *Vibrio fluvialis* (ATA-Vfl), a single amino acid mutation, L56V, improved selectivity increasing diastereomeric excess (de) from 14 to 89% compared to WT (Skalden et al., 2015). First, the residue Leu56 was identified from the docking model. The distribution of residues at this position in amine transaminases (ATAs) was analyzed using the 3DM database, a powerful tool for multiple sequence alignment (Kuipers et al., 2010). Only six amino acids were selected to develop a small mutant library (Skalden et al., 2015). Furthermore, even without a docking model, sequence alignments identify key function determining residues. Sclareol synthase from *Salvia sclarea* (SsSS), a class I terpene synthase, catalyzes hydroxyl group insertion. Three key sites (N431, S433, and T436) were selected from sequence alignment with other family members. Mutating these sites changed the water molecule nucleophile position in the active site, allowing it to attack the double bond from differing directions, selectively producing different stereoisomers (Jia et al., 2018).

### 2.2 Mutating Residues Involved in Direct Enzyme–Substrate Interactions

**FIGURE 1:**
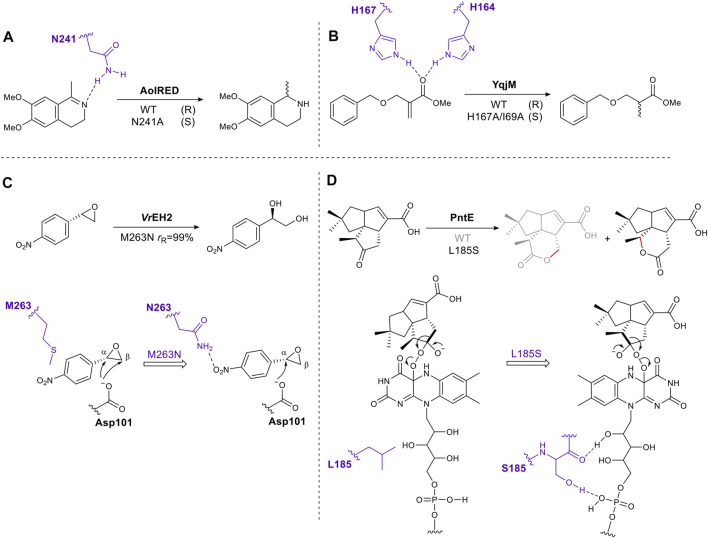
Schematic representation of rationally engineered residues directly involved in substrate binding tune reaction selectivity. **(A)**. Mutation to remove a hydrogen bond, altering the stereoselectivity of an imine reductase. **(B)**. Mutation of an active site histidine residue to remove a substrate hydrogen bond alters stereoselectivity of the ene-reductase, YqjM. **(C)**. Introduction of an enzyme-substrate interaction to alter regioselectivity (*r*_R_, ratio of regioisomers produced as a result of attack at α and β positions, producing R stereochemistry in each case). **(D)**. Mutation introducing enzyme-cofactor interactions changes regioselectivity of a Baeyer-Villiger oxygenase, PntE. Key residues are shown in purple.

Through inspection of the enzyme–substrate complex or substrate docking model, amino acids which interact directly with a substrate can be identified. Mutating these residues can eliminate or reduce affinity for a substrate by removing hydrogen bonds, electrostatic or hydrophobic interactions. However, if done carefully this approach can succeed in shifting the orientation of the substrate in the active site, thus altering the direction of reaction (e.g., hydride addition) or on which atom the reaction occurs, giving different stereoselectivity or regioselectivity respectively. For example, after examining the crystal structure of the imine reductase (IRED) AolRED, an N241A mutant was created to interrupt the interaction between the imine nitrogen atom and Asn241, moving the imine toward NADPH, thus changing the enantioselectivity (Aleku et al., 2016) (Figure 1A). In the flavin-dependent enzyme, YqjM, from the OYE family, two histidines act as hydrogen bond donors for the carbonyl oxygen of the α, β-unsaturated ester. Disrupting this interaction by mutating His167 to Ala, allowed the substrate to rotate and switched the stereochemistry of the product (Rüthlein et al., 2015) (Figure 1B).

Conversely, introducing hydrogen bonds through single amino acid mutagenesis can fine-tune substrate-binding positions as exemplified by the epoxide hydrolase VrEH2 (Figure 1C). Asn was introduced at position 263, moving the β carbon away from the catalytic Asp101, thus improving regioselectivity to 99% (Li F.-L. et al., 2018). A final example is PntE, which catalyzes Baeyer-Villiger oxidation in the biosynthesis of pentalenolactone D (Figure 1D). The flavin-dependent Baeyer-Villiger monooxygenases catalyze Baeyer-Villiger oxidations by using molecular oxygen to create a reactive peroxide species. This transformation, ketone to ester, is common in synthetic chemistry. The analogous chemical reaction uses organic peracids, e.g., mCBPA (meta chloroperbenzoic acid) which can be explosive. The oxygen atom is inserted adjacent to the carbonyl group via nucleophilic attack by the peroxide on the carbonyl, followed by rearrangement via a Criegee intermediate to afford the ester or lactone product (Liu et al., 2020). When a single mutation, L185S, was introduced into PntE a complete change in the regioselectivity of the reaction was observed. It was proposed that the serine side chain forms a hydrogen bond with the FAD (flavin adenine dinucleotide)-binding motif, altering the Criegee intermediate conformation, and switching the carbon that migrates (Chen et al., 2016) (Figure 1D).

### 2.3 Rational Mutagenesis to Substrate Binding Pockets to Alter Regio- and Stereoselectivity

Many enzyme families have highly defined, hydrophobic substrate binding sites that lend themselves to rational mutagenesis, both to increase substrate tolerance and change the selectivity of the enzyme. Bulky hydrophobic residues that line an enzyme active site are attractive targets for rational enzyme engineering. Modification of these residues can markedly reshape the substrate-binding pocket, usually creating more space and altering the hydrophobicity. Just enlarging the pocket can give rise to multiple substrate binding orientations which can lead to a loss of selectivity and multiple regio or stereoisomers. Thus, a balance is required between increased space to allow for more promiscuous substrate tolerance and ensuring the binding of specific orientations to maintain selectivity.

**FIGURE 2:**
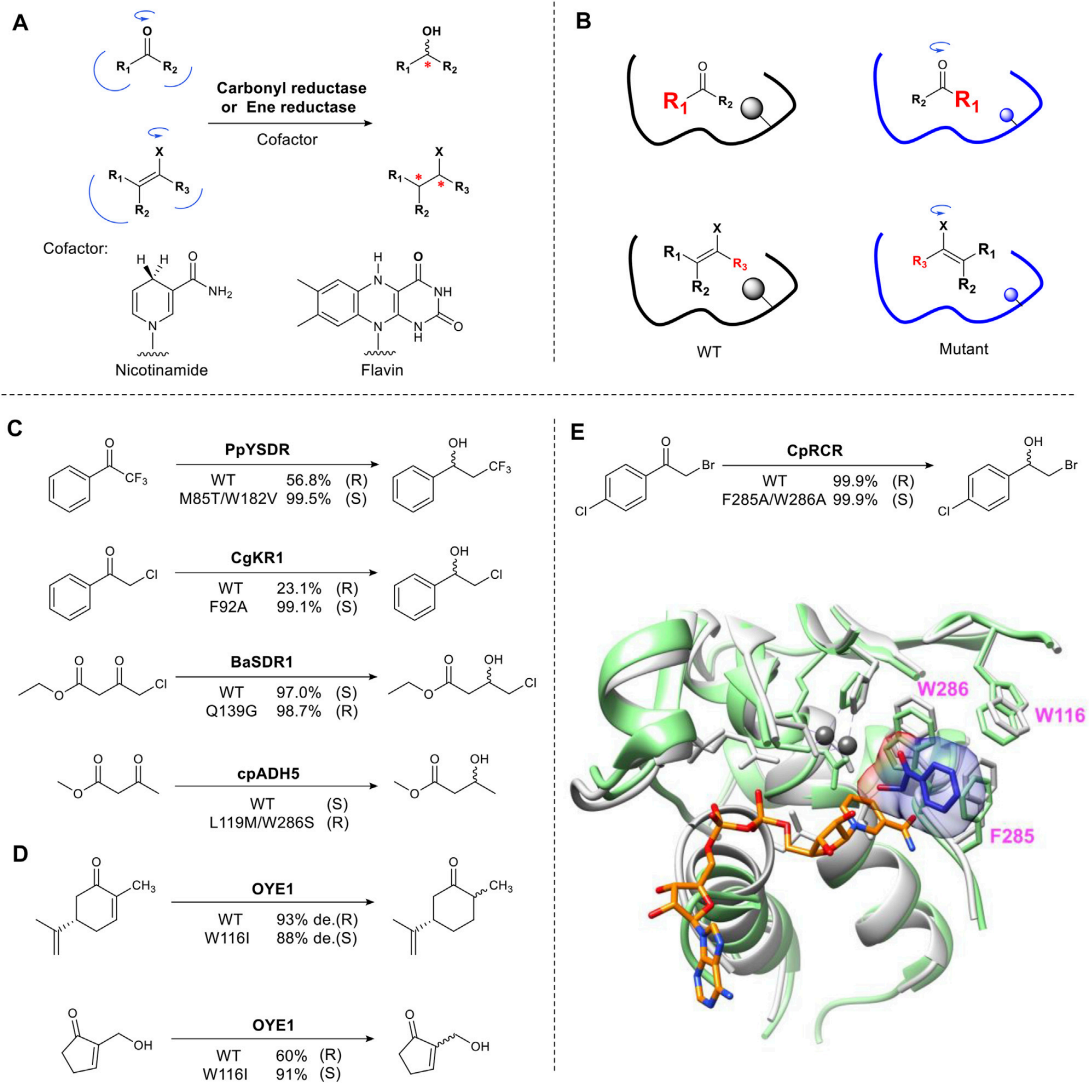
Reshaping the substrate-binding pocket flips substrate binding. **(A)** Carbonyl and ene-reduction are catalyzed by carbonyl reductase and ene reductase in the presence of cofactor. X = electron-withdrawing group. **(B)** Schematic representation of reversing stereoselectivity of enzymatic reduction using mutagenesis. **(C)** Examples of inverted stereoselectivity introduced by site-directed mutation in carbonyl reductases. **(D)** Examples of inverted stereoselectivity introduced by site-directed mutation in ene reductases. **(E)** Overlay of CpRCR crystal structure. Wild type CpRCR bound to NAD^+^ (orange) shown in grey (PDB: 3WLE) and H49A CpRCR bound to substrate hydroxyl phenylethanone (blue) shown in light green (PDB: 3WNQ). Zinc ions shown in dark grey (Wang et al., 2014). Bulky W286 and F285 in small binding pocket means only a small hydroxyl group can be accommodated.

This approach is exemplified in biocatalyzed stereoselective reduction reactions. Alcohol dehydrogenases and ene-reductases, catalyze the asymmetric reduction of carbonyl groups and activated carbon-carbon double bonds, respectively (Toogood and Scrutton, 2014) (Koesoema et al., 2020). These enzyme families have been studied extensively and are used in industrial biocatalysis (de Gonzalo and Paul, 2021) (Toogood and Scrutton, 2018). The mechanism involves the formation of an sp^3^ center from a prochiral sp^2^ carbon, creating a new stereocenter (Figure 2A). The enzyme binding pocket contains two sites that selectively bind the groups on either side of the carbonyl carbon in a *pro*-S or *pro*-R orientation, thus ensuring stereoselectivity. The typical approach for the rational redesign of these sites is to replace one or two large residues (Phe, Trp) located in the small binding pocket and mutate them to a smaller residue (Ala, Gly) resulting in “flipped” substrate binding e.g. from *pro*-R to *pro*-S or vice versa (Figure 2B). In practice this results in the hydride approaching the substrate from the opposite face (*Re* or *Si*) of the plane of the carbonyl, thus changing the enantiomer formed. Several alcohol dehydrogenases (ADHs) have been engineered to change their stereoselectivity including PpYSDR (Li et al., 2016), CgKR1 (Qin et al., 2016), CpRCR (Wang et al., 2014) (Nie et al., 2018), BaSDR1 (Li et al., 2021) and cpADH5 (Ensari et al., 2017) which catalyze asymmetric reduction of various α-halogen-substituted acetophenones and β-ketoesters (Figures 2C,E). Optimization of the reversed enantioselectivity can be achieved by further enlarging the binding pocket (Qin et al., 2018).

The well characterized flavin-dependent ene-reductase biocatalyst, old yellow enzyme family (OYE), catalyzes the reduction of α, β-unsaturated ketones. Mutating the hotspot Trp116 results in reversed stereoselectivity of OYE1. This was first reported using (S)-carvone as a substrate (Padhi et al., 2009) (Figure 2D). OYE1 can also accept numerous Baylis-Hillman adducts to afford complementary chiral β-hydroxy carbonyl compounds (Figure 2D) (Walton et al., 2011). Mutating Trp116 to a small residue such as Ala, flipped substrate binding, reversing the stereoselectivity. Introducing a large residue (Phe) did not affect stereoselectivity (Pompeu et al., 2013). For the reduction of Baylis-Hillman adducts, W116I not only provides more space for the substrate but also for a water molecule, which stabilizes the alcohol group of substrates through hydrogen bonding (Pompeu et al., 2012).

**FIGURE 3:**
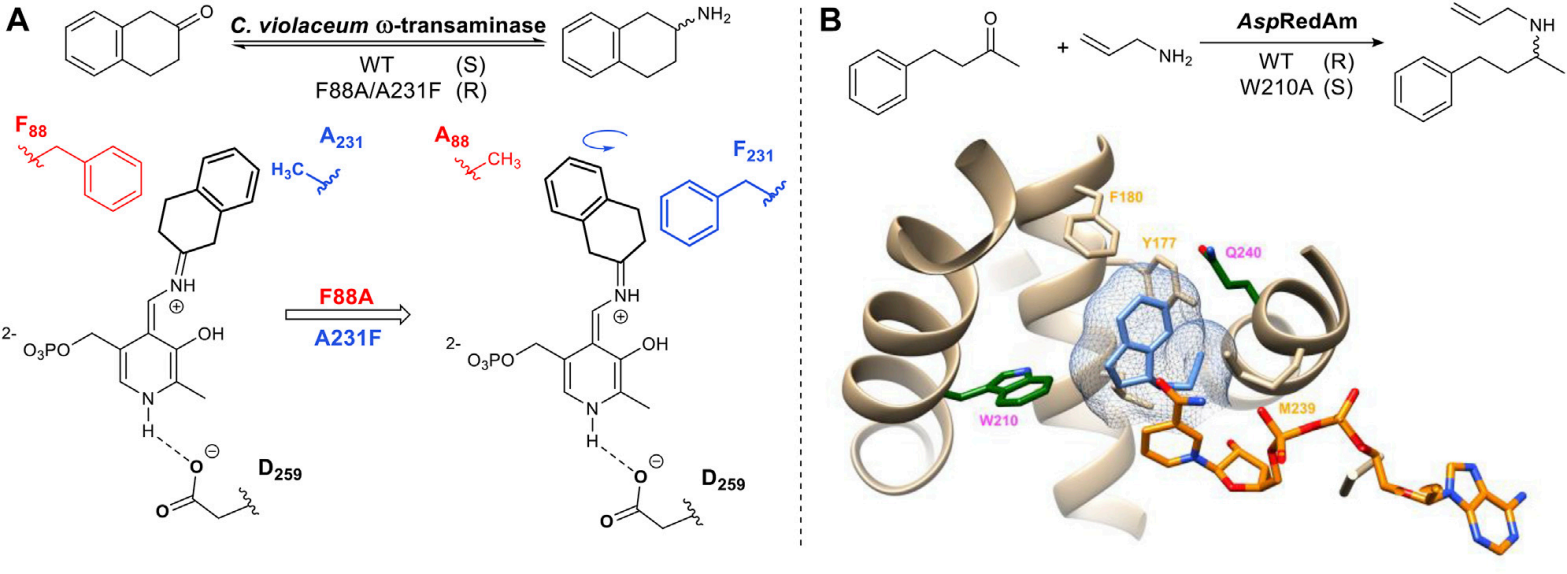
**(A)** Example of stereo-complementary transamination reaction and schematic representation of its reversed conformation, induced by mutation. The intermediate is formed via imine (Schiff base) formation of the ketone with the pyridoxyl phosphate cofactor. Stereoselective protonation of the Schiff base leads to a chiral amine product. The amine donor in this reaction is alanine. **(B)** An example of AspRedAm-catalyzed reductive amination. AspRedAm crystal structure (PDB: 5G6S) (Aleku et al., 2017), NADP(H) shown in orange and product rasagiline shown in light blue. Mutation of Q240 and W210 (dark green) can enhance and reverse stereoselectivity.

The above mentioned strategy has also been applied to the pyridoxyl phosphate-dependent transaminases. Humble *et al* utilized homology models of a transaminase from *Chromobacterium violaceum* allowing the rational design of the aldehyde/pyridoxal phosphate binding site to successfully switch the stereoselectivity of the reaction (Figure 3A) (Humble et al., 2012). Imine reductases (IRED) catalyze the same overall reaction to transaminases but differ in their mechanism of action. Imine reductases are NAD(P) H-dependent enzymes that catalyze the stereoselective addition of hydride to an imine (Figure 3B). For the imine reductase *Asp*RedAM, mutating key substrate interacting residues identified from the crystal structure e.g. W210 resulted in a complete switch in stereochemical outcome. Trp and Gln are located on either side of the chiral amine substrate. Mutants W210A and Q240A not only switched the stereochemistry of the product but also improved the stereoselectivity from 30% ee to greater than 90% ee (Aleku et al., 2017) (Aleku et al., 2018).

**FIGURE 4:**
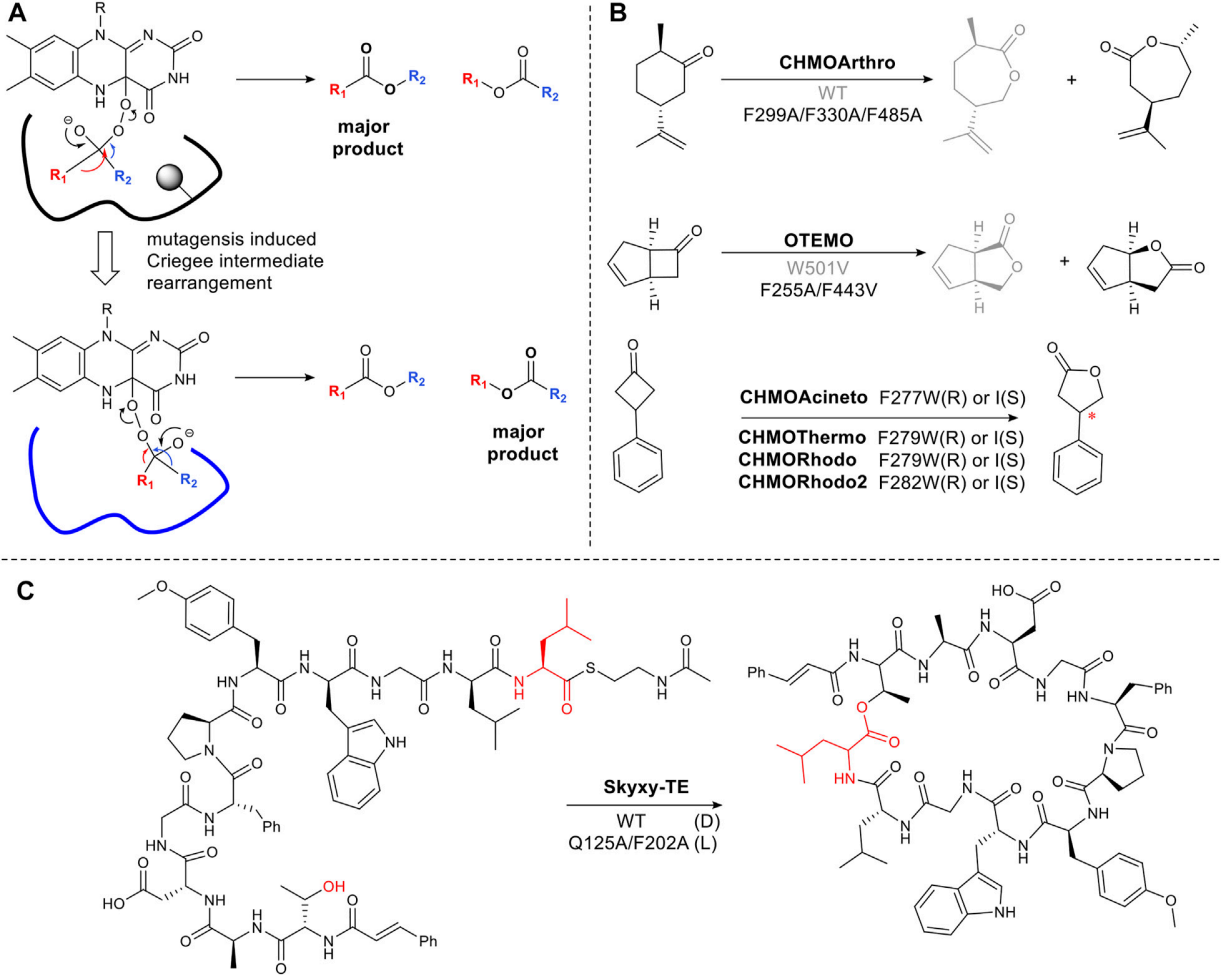
Steric hinderance control reaction selectivity. **(A)** Origin of selectivity in Baeyer Villiger reactions showing how a sterically bulky residue (shown in circle shadow) in the active site affects the orientation of the Criegee intermediate. **(B)** Examples of Baeyer-Villiger reaction catalyzed by BVMOs, **(C)** Bifunctional thioesterase (Skyxy-TE) catalyzing epimerization and cyclization of linear Skyllamycin analog, the wild type containing large residues Q125 and F202, only accepted the epimerized linear peptides.

As described in Section 2.2 flavin-dependent Baeyer-Villiger monooxygenases catalyze the transformation, ketone to ester or lactone (Liu et al., 2020). The regioselectivity of chemical Baeyer Villiger oxidations can be predicted based on the migratory ability of the carbons alpha to the carbonyl carbon. This is determined by the capacity of the carbon to stabilize the partial positive charge which develops in the transition state via the inductive effect. Thus tertiary carbons migrate in favor of primary carbons (Li G. et al., 2018). However, enzymatic reactions can result in unexpected Baeyer Villiger products due to the influence of the binding site on the transition state, favoring an alternative rearrangement of the Criegee intermediate (Figure 4A). Changing the steric bulk and hydrophobicity of residues in the binding pocket can thus result in the inversion of regioselectivity. For example, cyclohexanone monooxygenase from *Arthrobacter* sp. (CHMOArthro) catalyzes the oxidation of *trans* dihydrocarvone to a lactone (Figure 4B). The regioselectivity was reversed by the substitution of three phenylalanine residues with alanine, resulting in a change in substrate binding in the enlarged pocket (Figure 4B) (Balke et al., 2016). The same strategy was used for trimethylcyclopentenylacetyl-CoA monooxygenase (OTEMO) (Balke et al., 2017) and CHMO_Phi1_ (Messiha et al., 2018) (Figure 4B). Surprisingly, a single phenylalanine residue determines selectivity among CHMOs. Mutagenesis of this conserved phenylalanine close to the binding pocket in CHMOs predictably changes the regioselectivity of lactone formation (Hu et al., 2020).

The approach of targeting large hydrophobic residues and mutating them to reduce the steric bulk and hydrophobicity in an active site, can hold for a variety of enzymes even on large complex molecules. Skyxy-TE, a single thioesterase domain from a multi-modular, non-ribosomal peptide synthetase megaenzyme, catalyzes epimerization and cyclization of a linear peptide (Figure 4C). The epimerization is a highly unusual reaction for thioesterases which typically catalyzes either macrocyclization or hydrolysis. However, the small space around a conserved serine residue which covalently binds the linear peptide as an ester, constrains the intermediate in a *pro-R* configuration. Expanding the space, via mutation to Ala, allowed L-configuration (Yu et al., 2021) (Figure 4C).

The cytochrome P450s TxtE and 5NTSlav can perform direct regioselective nitration of L-tryptophan producing 4-nitro-L-tryptophan and 5-nitro-L-tryptophan respectively, using oxygen and nitric oxide as co-substrates (Barry et al., 2012) (Dodani et al., 2014). Nitration is a highly unusual reaction for a cytochrome P450, which typically carries out monooxygenation reactions e.g. hydroxylation, epoxidation as described in Section 2.1. The complementary regioselectivity of the reactions of TxtE and 5NTSlav is also intriguing. Dodani *et al*. identified that in TxtE His176 is involved in a direct edge-to-face interaction with the substrate, L-tryptophan. A single mutation of this residue in the flexible F/G loop of TxtE simultaneously controls loop dynamics and completely shifts the enzyme’s regioselectivity from the C4 to the C5 position of L-tryptophan. The resulting mutants TxtE-H176F; TxtE-H176Y and TxtE-H176W (this is the residue found in 5NTSlav) had also an increased affinity for L-tryptophan (Dodani et al., 2014). The larger side chains of these amino acids lead to a more tightly packed active site with different interactions with water molecules that shift the position of the substrate toward the heme, placing C-5 closer to the nitrogen of the proposed ferric-peroxynitrite catalytic intermediate (Barry et al., 2012) (Dodani et al., 2014) (Louka et al., 2020). Interestingly, the structure of TxtE does not significantly differ from that of conventional hydroxylating P450s and it is unclear why it carries out nitration and not hydroxylation. In fact, despite many decades of study it is not possible based on sequence or tertiary structure to predict what chemistry a particular P450 will catalyze.

### 2.4 Rational Mutagenesis to Expand Substrate Tolerance

**FIGURE 5:**
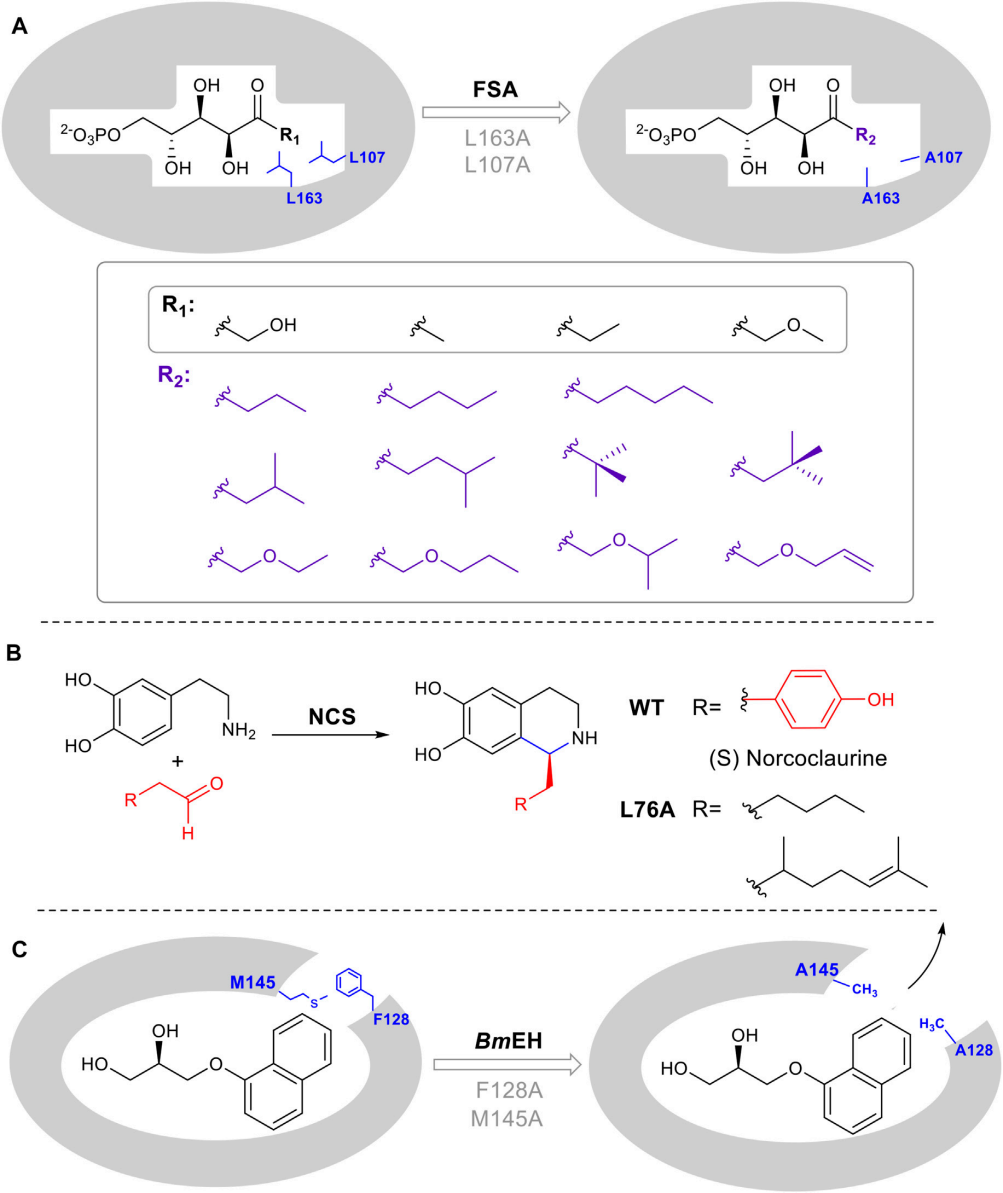
Enlarging active site augments substrate promiscuity. **(A)** D-fructose-6-phosphate aldolase (FSA) accommodates D-Fructose 6-Phosphate in the active site. Mutation of L107 and L163 to Ala, increases the pocket volume. **(B)** Norcoclaurine synthase catalyzes a Pictet-Spengler cyclization. L76A point mutation guided by mechanistic and computational studies, leads to improved substrate promiscuity. **(C)** Epoxide hydrolase BmEH M145 and F128 block the substrate tunnel. Mutation increases the size and improves the rate of product release.

While enzymes are attractive as catalysts due to their inherent stereo and regioselectivity, this often comes hand in hand with limited substrate tolerance which constrains potential biocatalytic application. There are several commonly used strategies to rationally expand and introduce promiscuity including enlarging the substrate-binding pocket, tuning the properties of a substrate tunnel or any conformationally flexible loops involved in substrate binding. Positions for mutagenesis can be identified using a crystal structure or homology model, followed by site-directed mutagenesis. For example, aldolases that catalyze the reversible, synthetically important condensation of aldehydes and ketones, are often limited by restricted recognition of donor components. Mutants L107A and L163A were designed in D-fructose-6-phosphate aldolase (FSA) from *Escherichia coli,* to accept a series of substrates containing various aliphatic side chains (Güclü et al., 2016) (Figure 5A). Additionally, in the computationally designed aldolase RA95.5-8F, a single amino acid mutation F112L makes the enzyme active toward large bulky cyclopentanone (MacDonald et al., 2020). In the epoxide hydrolase from *Bacillus megaterium* (*Bm*EH), mutagenesis on two predicted hotspots M145 and F128 enhanced enzyme activities of 6- to 430- fold toward various bulky substrates (Kong and Ma, 2014). A similar structure/computational guided approach was taken for norcoclaurine synthase (NCS). NCS is a plant alkaloid biosynthetic enzyme that catalyzes a stereoselective Pictet-Spingler condensation to produce pharmaceutically important tetrahydroisoquinolines (Figure 5B) (Lichman et al., 2015). A combination of structural and computational studies helped to elucidate the conformation of substrates in the NCS active site. A single leucine residue in the active site was identified as interacting with the substrate aldehyde R group. Once mutated, the change in the binding pocket made it possible to diversify the aldehydes tolerated by the enzyme (Lichman et al., 2015).

In several enzyme families, the substrate must pass through a tunnel connecting the buried active site with the enzyme surface. The tunnel is thus substrate selective and altering its properties can result in a broadening of substrates used by the enzyme, thus increasing promiscuity. *Bm*EH an epoxide hydrolase, showed poor activity toward bulky α-naphthyl glycidyl. The introduction of M145A or F128A expanded the tunnel, enabling product release. The activity was increased 42 and 25 times respectively (Figure 5C) (Kong and Yuan, 2014).

### 2.5 Mutating Residues Directly Involved in Catalysis

**FIGURE 6:**
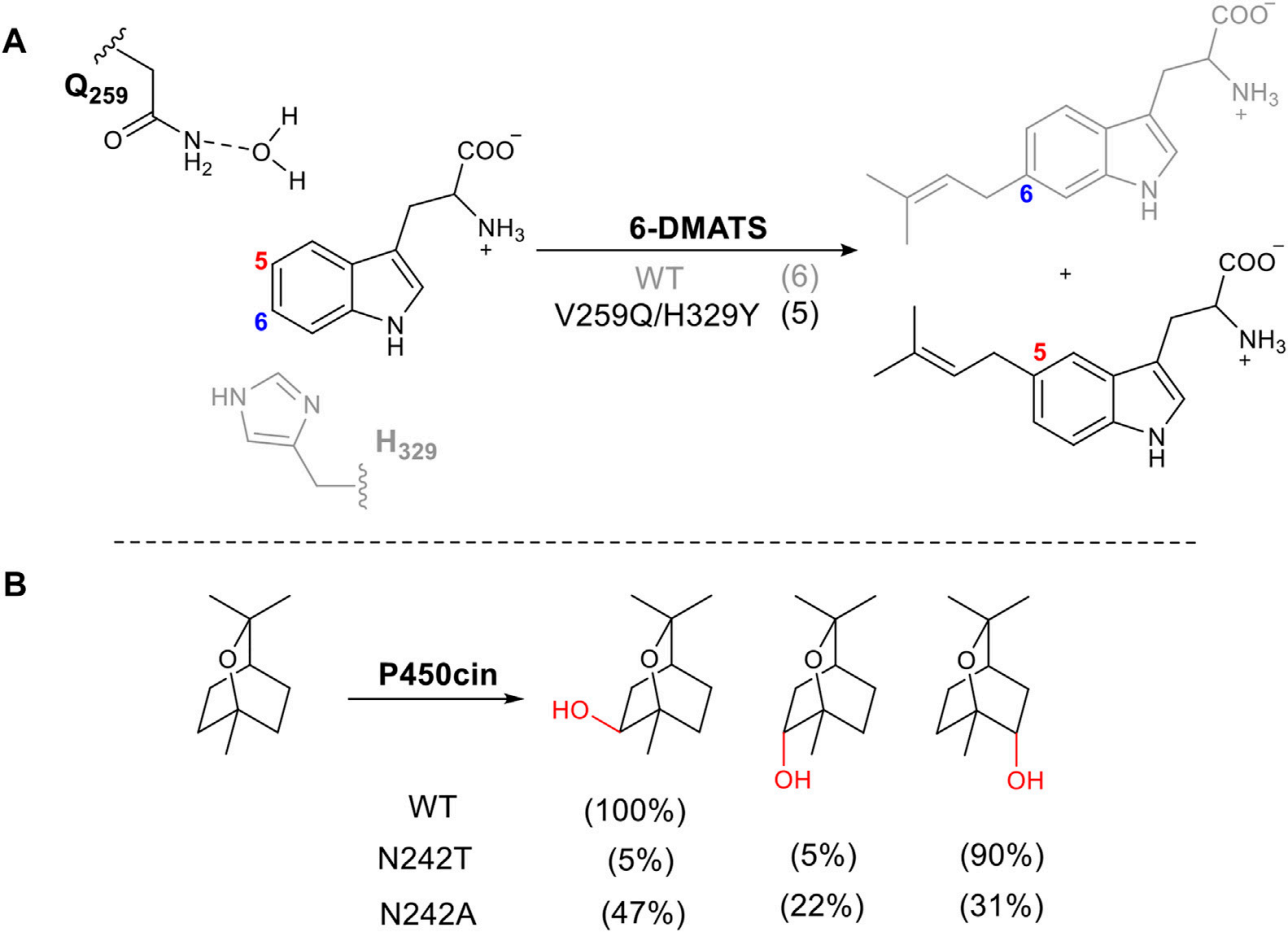
Tuning regioselectivity through altering catalytic residue. **(A)** 6-MATS catalyzes prenylation of tryptophan, natural selectivity shown in grey. **(B)** Effect of mutation of I-helix on P450cin hydroxylation.

Amino acids directly involved in catalysis are usually not the first choice for mutation when attempting to alter stereo- or regioselectivity, as it tends to result in loss of activity or negative changes in kinetic parameters. However, there are several examples where this approach has been successful. Bacterial tryptophan C6-prenyltransferases (6-DMATS) catalyze selective alkylation at C6 of the tryptophan indole ring. The reaction occurs via Friedel-Crafts alkylation using dimethylallyl diphosphate as the prenyl donor (Figure 6A). A double mutant V259Q/H329Y changed the catalytic base from His329, adjacent to carbon 6 to Gln259. This changed the regioselectivity of deprotonation via a structured water molecule, redirecting the prenylation to carbon 5. This approach was informed by structural elucidation and comparison of 6-DMATS and the complementary C5-prenyltranserase, 5-DMATS, allowing structure-guided engineering. The authors also note the challenges in this work in which several mutants lose selectivity and produce multiple regioisomers (Ostertag et al., 2021).

The cytochrome P450, P450cin, catalyzes an enantiospecific hydroxylation of monoterpene, 1,8-cineole, to produce (1R)-6-hydroxycineole (Figure 6B). P450cin contains a deviation from a P450 key motif (Stok et al., 2019). The I -Helix of P450s lies above the heme cofactor and contains the consensus sequence (AGXD/ET) which introduces a kink in the helix (Poulos, 2014). The threonine plays a vital role in the catalytic cycle, enabling the protonation of key intermediates in the reduction of oxygen to form the active oxidizing species (Nagano and Poulos, 2005). The residue in this position in P450cin is asparagine instead of threonine. When N242 is mutated to the consensus residue, threonine, the orientation of the substrate in the active site is changed, exposing a different C-H bond to hydrogen abstraction. The result is a dramatic alteration in regio and stereoselectivity (Figure 6B).

Alteration of catalytic residues can also confer new reactivity on enzymes, as exemplified by a redesigned pyruvate decarboxylase, *Zm*PDC which catalyzes asymmetric carbon-carbon bond formation (Meyer et al., 2011). Wild type *Zm*PDC uses Glu473 as a proton donor to enable the formation of a carbanion intermediate in the decarboxylation catalytic cycle releasing acetaldehyde. Replacing Glu473 with Gln, as well as providing benzaldehyde as an acceptor, enables the synthesis of phenylacetylcarbinol, thereby constructing a new C-C bond and a stereocenter (Meyer et al., 2011). Intriguingly, further study on Glu473 enabled precise control of its stereoselectivity by replacing it with larger or smaller residues (Wechsler et al., 2015).

## 3 Structure-Guided Semi-Rational Engineering

The examples above show that adjusting steric constraints in the binding pocket and changing key residues involved in catalysis or substrate binding, are powerful strategies to alter reaction outcomes. However, interactions between the substrate and the enzyme are often challenging to predict, and the steric modification strategy is substrate-dependent. For example, with carbonyl reductases, this strategy still limits substrates to an aromatic ring and a short-carbon chain on either side of the carbonyl carbon (Li et al., 2021). Structure-guided semi-rational engineering, through mechanistic understanding and computational modeling or more extensive screening of mutants, to identify key residues, are valuable additional strategies (Wang, Li and Reetz, 2017).

### 3.1 Computational Modelling

Computational tools are increasingly important in protein engineering. Free and easy-to-use software now exists to enable researchers to carry out bioinformatic analysis, create homology models and dock substrates into active sites. The power of these tools is apparent in the examples given above. However, these strategies do not account for protein dynamics affecting substrate binding/release or other aspects of the catalytic cycle. More powerful computational modeling such as Quantum Mechanics/Molecular Mechanics (QM/MM) methods, molecular dynamics (MD), quantum chemical (QC), and empirical valence bond (EVB) calculations are required to create a more nuanced understanding of the relationships between protein structure and function (Derat and Kamerlin, 2022). These techniques are widely used to enable the interpretation of experimental data to understand enzymatic mechanisms and rationalize the enzyme selectivity in several of the enzyme families discussed in this review including ene-reductases (Lonsdale and Reetz, 2015), diels-alderase (Byrne et al., 2016), BVMOs regioselectivity (Li G. et al., 2018) (Dong et al., 2022) and ketoreductase stereoselectivity (Serapian and Van Der Kamp, 2019).

A key attraction of computational methodology is the ability to carry out mutagenesis *in silico* and, thus, rationally redesign the enzyme, “preengineer”, prior to experiments. This can reduce experimental effort and generates key insights into the relationship between structure and mechanism. Empirical valence bond (EVB) calculates the reaction activation energy. This can be used to predict the effect on the catalysis of particular mutations. This approach allows the computational optimization of enzyme catalysis of a given reaction rather than predicting changes in selectivity (Mondal et al., 2020). Using, for example, Rossetta, enzyme specificity and substrate tolerance can be altered, by focusing on key areas of the active stite, introducing geometric constraints on areas that need to remain unchanged and analyzing the dynamic conformations to predict the outcome (Wijma et al., 2015). A recent example is AspB, an aspartase from *Bacillus sp*. YM55-1 that catalyzes the reversible deamination of aspartate. Wild-type AspB is highly substrate selective. A mutant of AspB, which demonstrated activity as a β-amino acid lyase, was only detected after cluster screening of 300,000 clones (Vogel et al., 2014). Recently, understanding of the enzymatic mechanism and structure of AspB was exploited to rationally engineer promiscuous variants for hydroamination. Engineering was carried out *in silico* using Rosetta Enzyme Design. This programme allows the selective redesign of particular parts of a binding pocket leaving key enzyme-substrate interactions untouched (Li R. et al., 2018). For example, four residues (Thr187, Met321, Lys324, and Asn326) which line the original aspartate, α carboxylate, binding site were replaced with hydrophobic residues to facilitate binding of the new α,β-unsaturated carbonyl electrophiles (Li et al.). Depending upon the penalty score of the constraints and structural inspection, just a few variants were identified and experimentally tested. Engineered AspB catalyzed hydroamination of various substituted acrylates (34 designs were screened for crotonic acid, 5 designs for (E)-2-pentenoic acid, 6 designs for fumaric acid monoamide and 30 designs for (E)-cinnamic acid). Moreover, the amine binding pocket of AspB was also engineered using the same strategy. A series of β-amino acids were thus synthesized with high regio- and enantioselectivity and on industrially relevant scales (Cui et al., 2021). These examples show how computational modelling can dramatically reduce experimental efforts.

Flexible loops around the active site play an important role in enzyme function but are often poorly defined in crystal structures. Molecular Dynamics (MD) simulations can identify key residues to influence substrate promiscuity. In the alcohol dehydrogenase, TbSADH, two rigid residues located on the loop near the binding pocket were identified from the analysis of the Root Mean Square Fluctuations (RMSFs) (Liu et al., 2019). Saturation mutagenesis of these residues was carried out to increase loop fluctuation. This generated a variant which could catalyze the reduction of a series of bulky ketones that are not accepted by the wild-type enzyme (Liu et al., 2019). Similarly, proline residues can provide structural rigidity and thus often play important roles in controlling protein structural dynamics. Mutating proline and its flanking residues located in loop regions near the active site can increase flexibility, thus indirectly adjusting the size of the binding pocket. For example, the TbSADH mutant P84S can accommodate non-natural bulky diaryl ketones, and mutant P84S/I86A carried out the reaction with a near-perfect conversion and stereoselectivity (Qu et al., 2021). In the case of phenylacetone monooxygenase (PAMO), the substitution of two conserved proline residues near the active site, increases the activity toward a series of 2-substituted cyclohexanones, which are not accepted by the wild-type PAMOs (Reetz and Wu, 2009) (Wu et al., 2010).

### 3.2 Structure Guided Semi-Rational Mutagenesis

Instead of subjecting one or two aminos acids to site-directed mutagenesis to alter selectivity, it is often necessary to investigate an entire loop or structural motif to identify the key residue or residues. This approach can generate many mutants but it is useful when there is no other obvious method to predict important residues or interactions. Common strategies include alanine scanning (mutating each residue to alanine to identify an effect) or saturation mutagenesis (mutating individual residues to all 20 proteinogenic amino acids to determine the most advantageous) (Wang et al., 2017). This methodology has been applied across diverse enzyme families catalyzing a variety of chemistries.

**FIGURE 7:**
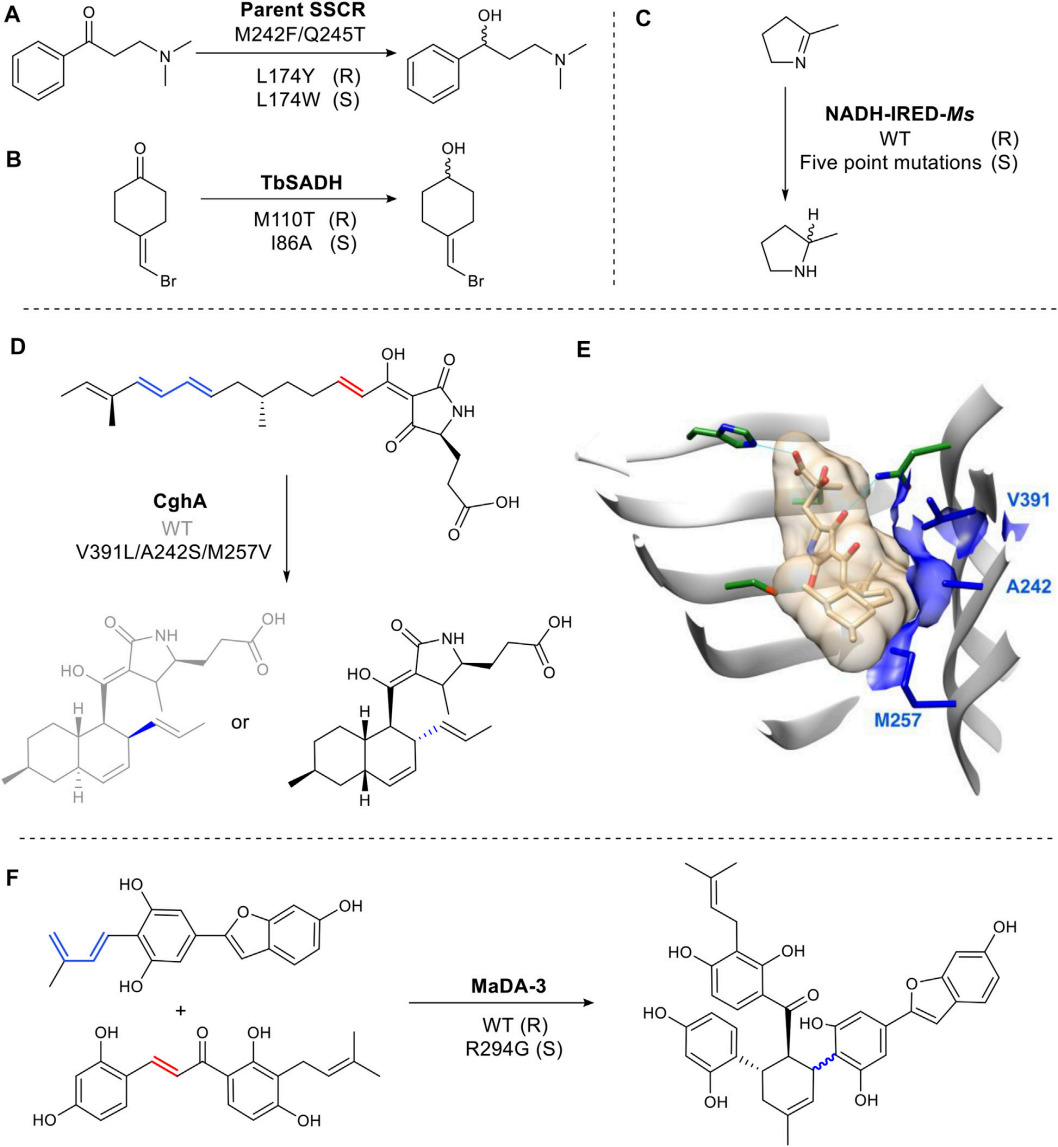
Examples of structure-guided engineering for altered steroselectivity. **(A)** Reductase SSCR reduces ketones to alcohols stereoselectively. **(B)** TbSADH is an alcohol dehydrogenase. **(C)** A multiple mutant of the imine reductase gives complementary stereochemistry to the WT. **(D)** Diels Alderase CghA WT and mutant produce different stereoisomers. **(E)** Crystal structure of CghA in complex with Diels–Alder adduct. Substrate binding residues involved in electrostatic interactions are shown in dark green. Hydrophobic interactions with the diene and dienophile and V391, A242, and M257 are indicated in blue. (PDB: 6KBC for the Cgh-1 complex structure, PDB: 6KAW for the apo CghA structure) (Sato et al., 2021). **(F)** Diels Alderase MaDA-3 WT and mutant produce different stereoisomers.

In the active site of the carbonyl reductase (SSCR) from *Sporobolomyces salmonicolor* AKU4429 (Figure 7A), a Tyr174 introduces a hydrogen bond between the tyrosine hydroxyl group and the substrate carbonyl oxygen resulting in the stabilization of *pro*-R conformation. While the mutant, Trp174, gave rise to the opposite orientation (Zhang et al., 2015) (Figure 7A). Reduction of 4-(bromomethylidene) cyclohexanone is catalyzed by wild-type TbSADH with a poor enantioselectivity (66% ee), as its highly symmetric structure can bind either a *pro*-R or *pro*-S conformation (Figure 7B). Mutating M110 to threonine increased R selectivity to 97% e.e. while mutation at I86 enhanced S selectivity to 98% ee. (Agudo et al., 2013). It was proposed that the side chain of Thr110 is responsible for the formation of a *pro*-R conformation by forming a hydrogen bond with a bromine identified through molecular dynamics simulations (Maria-Solano et al., 2017) (Figure 7B). The hydrogen bonding of the halogen atom and XH/π interaction have been shown on a molecular level to play a critical role in determining the stereoselectivity (Chen et al., 2018).

While rare, changes to the binding site of a cofactor can also be used change selectivity. For example, in the imine reductase NADH-IRED-Ms (Figure 7C), five amino acid mutations (positions 241- 245) change the orientation of NADH cofactor binding. The resulting switch in hydride attack, gives an inversion of product stereochemistry compared to wild type (Stockinger et al., 2021).

Over the last several years there has been interest in the discovery and characterization of Diels Alderases (DAs) (Minami and Oikawa, 2016), as well as the design of artificial enzymes for Diels–Alder reactions (Ghattas et al., 2021). A biocatalysed Diels Alder reaction is of significant interest as the chemical transformation often requires high pressures or temperatures and long reaction times (Yang and Gao, 2018). However, stereochemical control (endo/exo) of the enzymatic reaction is vital to make any future biocatalytic process viable. CghA catalyzes an intramolecular Diels–Alder reaction to form octalin natural products (Sato et al., 2021). The stereoselectivity was successfully converted from endo to unfavored exo-selectivity by reshaping the active site with three mutations to organize an exo transition state for the diene and the dienophile (Sato et al., 2021) (Figures 7D,E). *Morus alba* Diels-Alderase (MaDA) catalyzes an intermolecular Diels–Alder reaction. Interestingly, MaDA-3 has natural exo-selectivity. The transition state is stabilized through a cation-π interaction between a key R294 residue and the dihydroxylphenyl ring of the dienophile. Replacement of the residue afforded some endo product (Gao et al., 2021) (Figure 7F).

**FIGURE 8:**
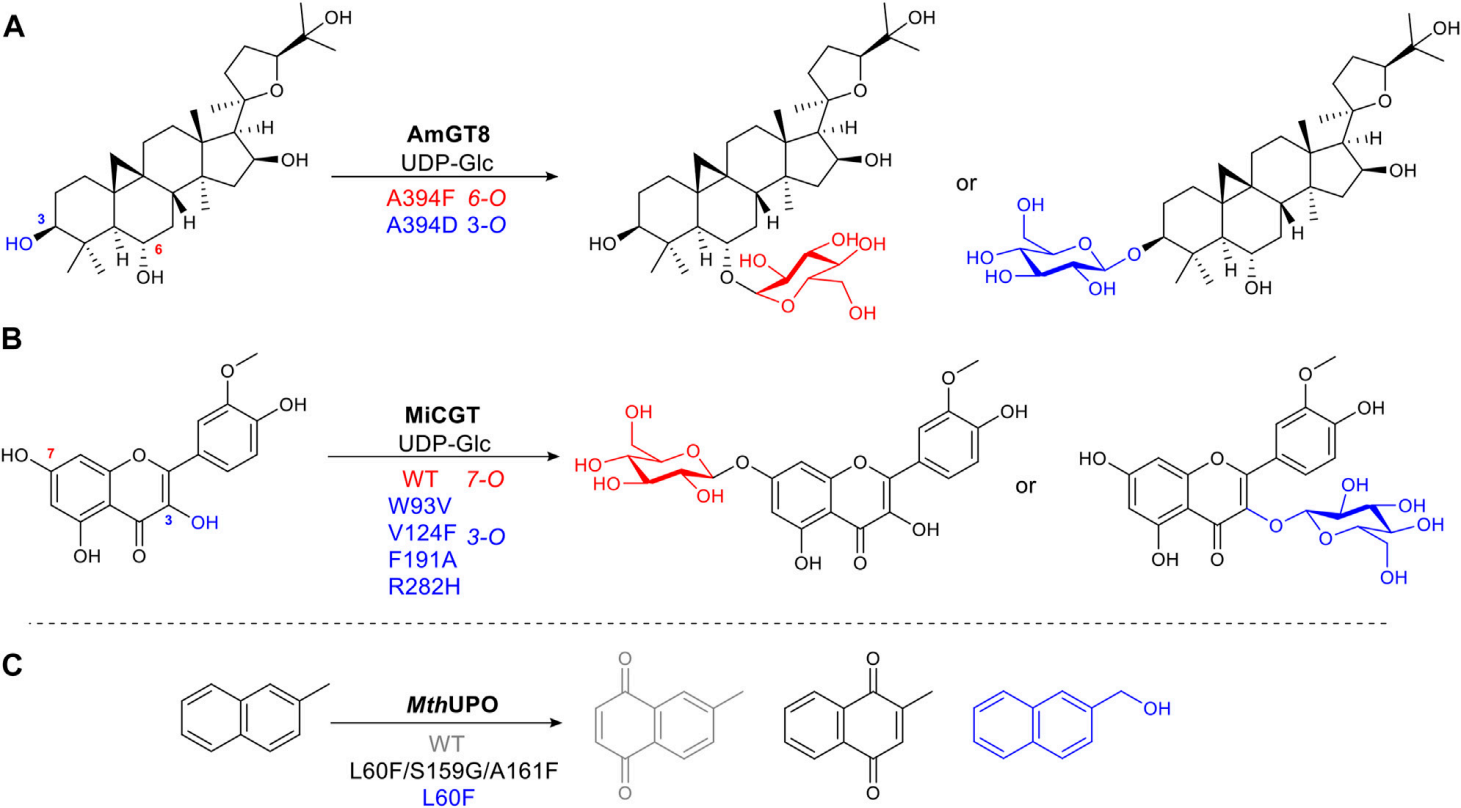
Examples of structure-guided protein engineering to alter regioselectivity. **(A)** Glycosyltransferase AmGT8 catalyzing triterpene 3-/6-O-glycosylation, a conserved residue at position 394 is important for regioselectivity. **(B)** 3-/7-O-Glycosylation of a flavonoid catalyzed by wild-type glycosyltransferase MiCGT and a VFAH mutant. **(C)** Heme-containing peroxygenase *Mth*UPO catalyzing aromatic oxidation. Mutations alter substrate binding position to afford diverse products.

Glycosylation is a common, vital, late-stage functionalization reaction in natural product biosynthesis (Kren and Martinkova, 2012) (Liao et al., 2018). Selective chemical glycosylation of complex molecules is challenging, often requiring tedious protection and deprotection steps. Thus a selective enzymatic route is desirable. Wild type glycosyltransferases (GTs) catalyze glycosidic bond formation, which suffers from poor regioselectivity due to its large binding pocket, and thus multiple substrate-binding modes or conformations (Nidetzky et al., 2018). Longer distances between substrate and enzyme make it difficult to determine in a rational way which sites affect the substrate binding position. To narrow this down without alanine scanning the whole site or using full saturation mutagenesis of each site, an approach known as “small but smart” was used. This less intensive method mutates the target residue to a representative of each class of amino acids, e.g. positively charged, negatively charged, polar uncharged etc., to identify which group makes the greatest impact, thus significantly reducing experimental effort. AmGT8 catalyzes glycosylation of triterpenes (Figure 8A). Activity assays using a selection of substrate derivatives were performed, followed by the comparison of different docking models of the substrate with desired regioselectivity. From the activity study of various size triterpenes, three AmGT8 docking models with different regioselectivities were established, and active site amino acids were divided into four groups. After screening a small set of mutants, the conserved residue, A394, was identified as crucial for regioselectivity (Zhang et al., 2021).

Alanine scanning the active site was utilised to change the selectivity of MiCGT, which catalyzes glycosylation of flavonoids. After iterative saturation mutagenesis, a mutant containing four amino acid changes, that shifted the regioselectivity from 7-O to 3-O, was identified (Wen et al., 2021) (Figure 8B).

The peroxygenase *Mth*UPO catalyzes the oxidation of 2-methylnaphthalene. Fine-tuning substrate position relative to the active oxygen species formed on the heme iron center, accomplished diverse chemo- and regioselective oxidation reactions. For mutant L60F, introducing a bulky phenylalanine allowed only the methyl group to get close enough to the catalytic center to be oxidized. Additional mutations, L60F/S159G/A161F, increased hydrophobicity, pushing the naphthyl aromatic ring moiety toward the heme-iron center, to afford a different quinone product (Knorrscheidt et al., 2021) (Figure 8C).

## 4 Future Challenges and Outlook

There is little doubt that biocatalysis will feature heavily in the future of synthesis whether it is bulk chemicals or complex molecules. Small precursor chemicals are frequently derived from fossil fuels and are thus unsustainable. Equally, there is a need for optically pure precursors and enzymes are well suited to their production. Complex, high-value molecules, represent excellent targets for biocatalysis, especially as the initial high cost of biocatalyst development may be more justifiable. A recent application of biocatalysis to the synthesis of the COVID-19 drug by Merck shows that there is already a recognition of the power of biocatalysis and a clear push from industry toward increased use (McIntosh et al., 2021).

Great strides have been made in both the understanding and engineering of enzymes and this is an exciting time in the field. This has been facilitated by several factors. Advances in molecular biology mean that processes such as cloning and mutagenesis are facile and can even be automated. Structural information from X-ray crystallography, NMR and now EM is vital for enzyme engineering, as demonstrated above. Developments in these techniques and data processing has speeded up the process of structure elucidation. Excitingly, AlphaFold now offers the opportunity to predict structures of structurally uncharacterized or indeed un-crystallizable enzymes (Jumper et al., 2021) (Varadi et al., 2022). Improved computational methods are providing greater insight into catalytic mechanisms and molecular dynamics. Developments in cofactor recycling and improving enzyme stability mean that enzymes previously thought of as too complex or those requiring expensive cofactors are now viable targets for use as biocatalysts (Mordhorst and Andexer, 2020).

Such progress has meant that the ambition of the field has also grown. The goal for many in the field is to redesign enzymes to carry out new reactions, particularly those chemistries not currently found in biology, so-called “new to nature” transformations. Some of the best examples of new to nature reactions have come via directed evolution as pioneered by Frances Arnold (Chen and Arnold, 1993). A classic example from the Arnold lab evolved P450_BM3_ to carry out cyclopropanation using a carbene generated *in situ* (Coelho et al., 2013)*.* While cyclopropanation reactions are not new to nature, they are new to P450s. In addition, the chemistry, using a diazoacetate to generate the Fe-bound carbene, does not have a known analog in biological chemistry (Liu and Arnold, 2021). This result has been built on in recent examples, including engineered cytochrome P450 catalyzed atom transfer radical cyclization (ATRC), where a new C-C bond and a new C-Halogen bond are formed (Zhou et al., 2021). In a further recent development from the Arnold lab, they report the P450 catalyzed enantioselective ring expansion of aziridines to azetidines (Miller et al., 2022). Other methods to enable new to nature reactions (Leveson-Gower et al., 2019) include utilizing expanded genetic code via amber codon technology and tuning active site electronics (Ortmayer et al., 2020) as well as using Rosetta to design enzymes to carry out new to nature chemistry (Crawshaw et al., 2021).

The integration of chemocatalysis and enzymes has also been pursued as a way of combining the advantages of chemical and enzymatic catalysis. The goal is to utilize proteins or enzymes as scaffolds to house small-molecule catalysts (Schwizer et al., 2018). The catalysts themselves are usually organometallic and carry out the chemistry that is rare or not found in nature. They effectively act as an unnatural cofactor, coordinated to a native or engineered protein scaffold. This delivers all the advantages of enzyme catalysis, e.g., hydrophobic environment, increase in the effective concentration of reactants and most importantly, stereo and regioselectivity, which are key challenges in organic synthesis (Schwizer et al., 2018). Scaffolds are chosen to be accessible to a broad range of substrates.

Despite this progress, significant challenges remain. Protein engineering either rationally or via directed evolution is time-consuming. Efficient screening of mutants usually requires operationally simple, robust spectrophotometric assays, that can be read on plate reader or similar. Creating such assays is in turn time consuming and in some cases may not be possible. Equally, screening for one output may miss other potentially useful data, e.g., changes in chemistry or selectivity. Detection of serendipitous outcomes requires more powerful analytics. High-throughput LCMS would provide more information, i.e, all reaction products including stereo- and regioisomers but it is more operationally complex and expensive. Additionally, major improvements in the analysis of high throughput LCMS data, which is orders of magnitude more complex than that from a colorimetric assay, are required.

As seen in examples throughout this review “preegineering” *in silico* is increasingly important. Improved methods for *in silico* screening, such as deep mutational analysis (Dunham and Beltrao, 2021), will reduce experimental effort and increase the rate of progress. The logical progression of these strategies is *in silico* enzyme design facilitated by, for example, Rosetta. This tool has already been applied by several groups (Crawshaw et al., 2021) (Basanta et al., 2020) (Richter et al., 2011). Both approaches will also generate greater insight into the factors affecting substrate binding and catalytic mechanism which in turn will lead to more predictability. (Bunzel et al., 2021) (Jindal et al., 2019).

Thus, there are many exciting routes from which new biocatalysts will emerge. What is clear, is a greater understanding of the relationship between protein structure and enzyme function is needed. While crystallographic data is crucial for our understanding of enzymes, they offer us a static picture of an enzyme in one state. Insight into protein dynamics during catalysis will enable a greater understanding of the roles of residues in the active site and beyond, including their cooperativity. The importance of flexible regions in enzyme catalysis, often poorly defined in crystal structures, has been poorly explored. Computational methodology will enable a greater understanding of the role of flexible regions and protein dynamics in catalysis. Thus new computational tools and their democratization will, alongside the ongoing protein engineering revolution, lead to the second great revolution in enzyme engineering and is already proving exciting (Büchler et al., 2022) (Crawshaw et al., 2021). This combination will provide a much-needed pool of sustainable catalysts and hopefully a new era in chemical synthesis.
